# The Administration of Drugs in Conjunction with Physical Methods in the Treatment of Disease

**Published:** 1906-08

**Authors:** W. T. Rushin

**Affiliations:** Resident Physician Vineville Sanitarium, Macon, Ga.


### THE ADMINISTRATION OF DRUGS IN CONJUNCTION WITH PHYSICAL METHODS IN THE TREATMENT OF DISEASE.

By W. P. RUSHIN, M.D.,

Resident Physician Vineville Sanitarium, Macon, Ga.

Unfortunately, the majority of practicing physicians to-day are deeply prejudiced against those who have introduced into their practice physical methods and physical paraphernalia in the treatment of disease. There are two paramount reasons for this. First, they have received instruction, while attending medical college only pertaining to drug prescribing and drug taking; second, charlatans have seen the financial benefit accruing to themselves in taking hold of many of these physical agents, notably electrical apparatus, and by their bold advertising and unscrupulous methods duped a long-suffering, drug-taking public. Nevertheless, neither of these reasons justify the stolid indifference and more hostile criticism with which the general profession view and hastily dismiss in judgment these physical agencies in the treatment of disease. Really, they both, i. e., drug prescribing and physical therapeutics, go. or should go, hand in hand. *Neither* are absolute specific in every disease—and indeed are in but very few—but used intelligently in conjunction with each other, the physician is far better equipped to cope successfully with a much larger number of diseased conditions. To illustrate: All well-informed physicians of to-day will admit that *massage* (either by hand or by the intelligent application of many of the vibratory machines on the market), hydro-therapeutics and electricity in conjunction with forced feeding and the administration of such drugs as iron, malt, strychnine, aloes, etc., as indicated, accomplish far better and more permanent results than the single administration of drugs alone. There is a well-recognized system in vogue among the profession which abundantly proves, and has proven, this. Again the true physician can best, of all other men, afford to be altruistic. Our great Master himself "went about doing good," and though divine as He was, as well as human, He did not hesitate to unite the *material* with the spiritual and physical in healing disease, for He anointed the eyes of the blind, gave the helping hand to raise the cripple, and even the dead. I would not be here misunderstood as endorsing or standing for "Christian Science," "Dowieism" and others of like cult *ad nauseum,* but I am pleading for a fuller and more equitable recognition by the profession at large of the long list of really useful physical agents that are ready to help us in doing our life work the very best.

Brethren, let us be honest with ourselves and the public, *investigate, educate, use* and apply *any* remedy that is based on physiology and pathology, and I submit that such remedies as electricity, hydrotherapy, light, heat, vibration, etc., stand just here.

*30 Hardeman Avenue.*

